# Antidepressant Drug Sertraline against Human Cancer Cells

**DOI:** 10.3390/biom12101513

**Published:** 2022-10-19

**Authors:** Diana Duarte, Nuno Vale

**Affiliations:** 1 OncoPharma Research Group, Center for Health Technology and Services Research (CINTESIS), Rua Doutor Plácido da Costa, 4200-450 Porto, Portugal; 2 Faculty of Pharmacy, University of Porto, Rua Jorge Viterbo Ferreira 228, 4050-313 Porto, Portugal; 3 CINTESIS@RISE, Faculty of Medicine, University of Porto, Alameda Professor Hernâni Monteiro, 4200-319 Porto, Portugal; 4 Department of Community Medicine, Health Information and Decision (MEDCIDS), Faculty of Medicine, University of Porto, Rua Doutor Plácido da Costa, 4200-450 Porto, Portugal

**Keywords:** anticancer activity, sertraline, antidepressant drugs, human cancer cell lines

## Abstract

The use of FDA-approved drugs for new indications represents a faster and more economical way to find novel therapeutic agents for cancer therapy, compared to the development of new drugs. Repurposing drugs is advantageous in a pharmacological context since these drugs already have extensive data related to their pharmacokinetics, facilitating their approval process for different diseases. Several studies have reported the promising anticancer effects of sertraline, both alone and combined, in different types of cancer cell lines. Here, we performed a literature review on the anticancer potential of sertraline against different human cancer cells, more specifically in lung, colorectal, breast, hepatocellular, leukemia, brain, skin, oral, ovarian, and prostate cancer. Taken together, these findings suggest that sertraline decreases cell viability, proliferation, migration, and invasion, induces apoptosis, and causes cell cycle arrest in different types of cancer cells, besides being an established P-glycoprotein modulator. It was also found that this drug is able to modulate autophagy, cause DNA fragmentation, and induce radical oxygen species (ROS) formation. Moreover, it was found this drug targets important cellular pathways involved in tumorigeneses such as the TNF-MAP4K4-JNK pathway, the antiapoptotic pathway PI3K/Akt/mTOR, and the AMPK/mTOR axis. This drug also interferes with the TCTP/P53 feedback loop and with the cytosolic free Ca^2+^ levels. Together, these results suggest that sertraline may be a promising compound for further evaluation in novel cancer therapies.

## 1. Introduction

Cancer is the second greatest cause of death in the United States (US) and continues to be one of the most significant public health issues worldwide [1]. The coronavirus disease 2019 (COVID-19) pandemic still has an impact on the most recent statistics on cancer incidence and mortality due to lockdowns that restricted access to medical care combined with the associated fear of COVID-19 exposure, which led to delays in diagnosis and treatment of oncological malignancies [2]. In 2022, the US is expected to have more than 1,900,000 new diagnostics of cancer and more than 600,000 cancer-related deaths, according to the American Cancer Society’s most current report on cancer statistics [1]. Specifically, the number of fatalities from lung cancer, which continues to rank top among all types of cancer for both sexes, is predicted to reach 350 per day. The second and third most often diagnosed cancers, breast and colon cancer, respectively, will account for 43,780 and 52,580 estimated fatalities in the US in 2022. Additionally, leukemia is expected to account for 60,650 new cases and 24,000 deaths, liver, and intrahepatic bile duct cancers to 41,260 new cases and 30,520 deaths, lymphoma to cause 89,010 new diagnosed cases and 21,170 deaths, pancreatic cancer to have 62,210 new cases and 49,830 deaths, and skin cancer (excluding basal and squamous) to account for 108,480 new cases and 11,990 deaths [1]. These data highlight the critical need for the development of innovative and more effective cancer treatments that can contribute to lower cancer patient mortality and enhance their quality of life while undergoing treatment.

The estimated costs for the de novo development of a single anticancer drug are about $650 million, in a process with a duration of approximately 17 years [3,4]. Moreover, only about 10 new anticancer drugs are approved every year by the Food and Drug Administration (FDA) [3,5]. Drug repurposing, also known as drug repositioning, is an alternative approach to the de novo drug development that re-investigates drugs that have previously received FDA approval and are available on the market for novel therapeutic purposes other than their original ones [3]. This can also happen when the therapeutic uses of a drug are expanded to treat additional diseases that are related to the one being treated, such as other types of cancers. Since the data related to their absorption, distribution, metabolism, elimination, and toxicity (ADMET), acceptable therapeutic doses, and safety information from clinical trials are already extensively described, this approach requires less time and money than the development of novel drugs de novo, shortening the process approval and boosting the likelihood of these drugs entering clinical trials [6,7]. Another advantage of drug repurposing is that most repurposed drugs can be taken orally with good tolerance; furthermore, since most of them are generic medicines, they are more affordable than newly patented drugs [8]. One must keep in mind that novel side effects that may not be described for their original indication can arise for a new therapeutic indication [3], making it necessary for clinical trials to further validate the application of these drugs in novel indications [3].

Over the years, the number of repurposed drugs has been increasing and different drugs have already been successfully repositioned against several diseases [9]. Thalidomide, for example, initially discovered as an anti-emetic drug, has already been approved for the treatment of leprosy and multiple myeloma [3,10]. Another drug, metformin, which was initially proposed for the treatment of diabetes, is now in clinical trials as an adjuvant drug for cancer treatment [3,11]. Even with the COVID-19 pandemic, drug repurposing was demonstrated to have an important role in accelerating the discovery of novel therapies for this disease. For example, the antiparasitic drugs chloroquine and ivermectin have been proposed for the treatment of COVID-19 [12,13,14,15].

Several studies have already suggested the positive outcomes of using central nervous system (CNS) drugs as adjuvant therapy to reduce depressive events and improve the quality of life in cancer patients [16,17,18]. Moreover, the repurposing of CNS drugs to treat different types of cancer has already been reported, and preliminary findings in human cancer cells and animal models suggest that this class of therapies may have potential anti-cancer effects [19,20,21,22,23].

**Figure 1:**
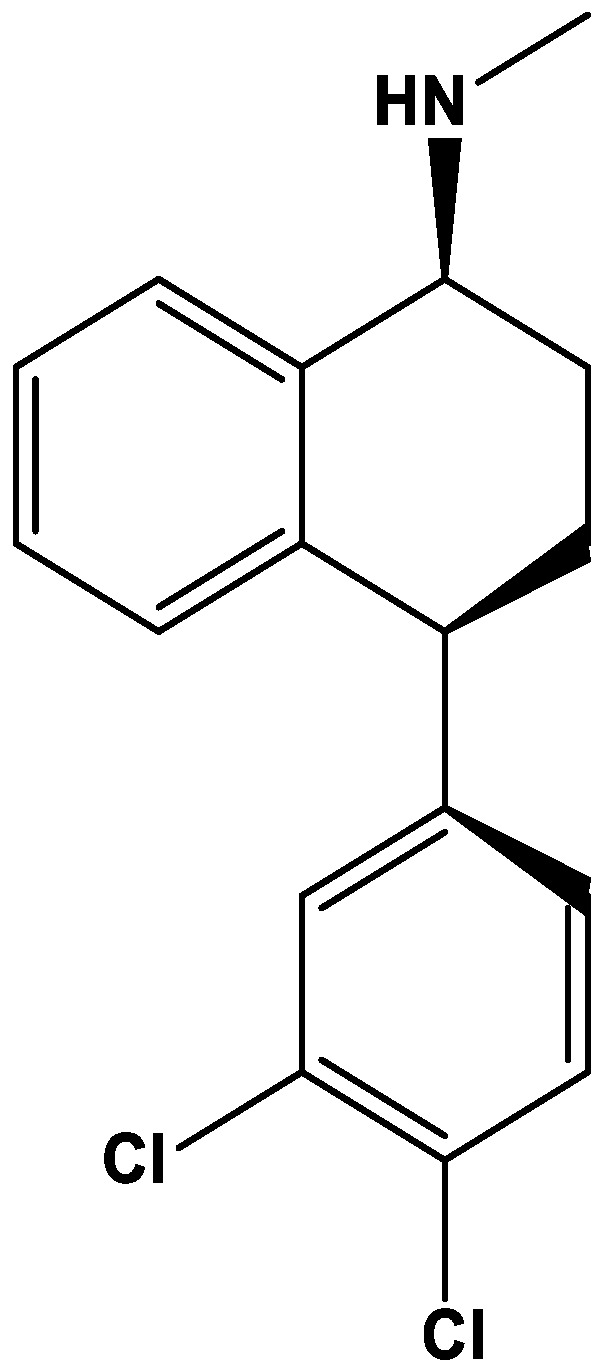
Chemical structure of sertraline. The structure was obtained using ChemDraw software (version 12.0, PerkinElmer, Inc. Waltham, MA, USA).

Sertraline (Figure 1), sold under the commercial name Zoloft^®^, is a drug belonging to the class of selective serotonin reuptake inhibitors (SSRIs), which are antidepressants commonly used in the management of psychiatric disorders [24]. This drug was among the first SSRIs compounds to be approved by the FDA for clinical use, in 1992 [25]. This drug demonstrates efficacy in the treatment of depression, including major depressive disorder, anxiety and eating disorders, and also premenstrual dysphoric disorders [25].

**Figure 2:**
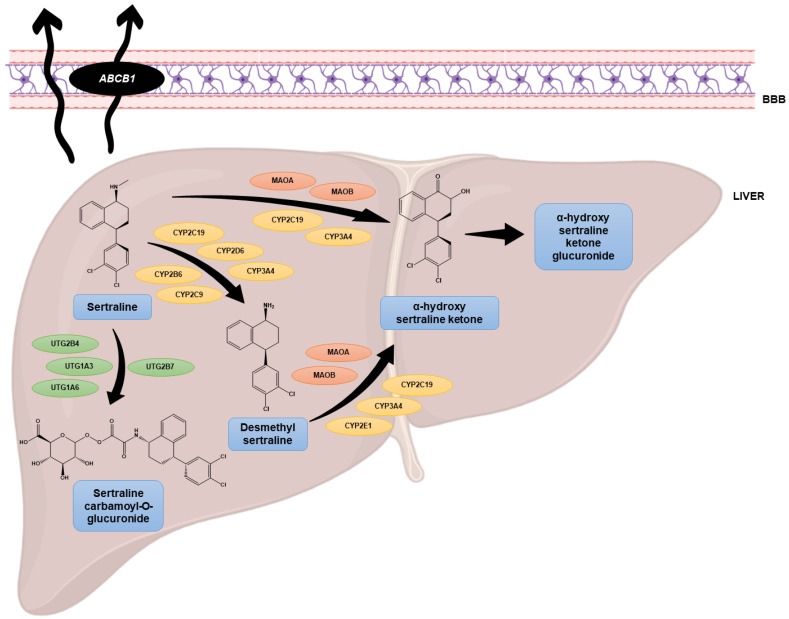
Metabolism of sertraline in the liver and its interaction with the BBB. Adapted from [28].

Compared to other antidepressant drugs, sertraline has an improved profile of tolerability, less lethality in cases of overdose, and a reduced risk of dependence [25]. Using an in vitro model of the blood–brain barrier (BBB), it was found that sertraline easily crosses this barrier [26]. Moreover, both sertraline and its active metabolite desmethylsertraline were found to interact with P-glycoprotein, a protein present in the BBB encoded by the *ABCB1* gene, with high affinity [27]. The main metabolism of sertraline occurs in the liver (Figure 2) and most studies have focused their attention on the demethylation of sertraline to desmethylsertraline, a reaction mediated by multiple cytochrome P450 (CYP) enzymes [28].

Although sertraline can target the CYP2D6 isoenzyme, it happens in a lower degree than other antidepressants, reducing the chance of negative drug–drug interactions, which is particularly important in elder patients receiving more than one drug [25]. Additionally, this drug has already been extensively studied and prescribed for clinical use, which supports its efficacy and favorable toxicological profile [25]. Its primary mechanism of action involves the inhibition of serotonin reuptake into the pre-synaptic nerve terminals, which causes the elevation of serotonin levels in the synaptic gap [29].

Despite its well-known benefits in the treatment of psychiatric disorders, several studies have already investigated the anticancer potential of SSRIs in different types of cancer cells [25]. For example, fluoxetine, an older SSRI than sertraline, has demonstrated the ability to decrease the proliferation of PC-3, DU-145 and LNCaP prostate cancer cells, both in vitro and in animal models [30]. In other studies, this SSRI has also been able to induce apoptosis of C6 glioma [31] and neuroblastoma cells [32], in a mechanism involving the activation of mitogen-activated protein kinase (MAPK) N-terminal kinase (JNK) pathway, including phosphorylation of the c-Jun protein [32]. Another study observed a decrease in the cell viability of Jurkat malignant T cells after treatment with 20 µM of fluoxetine, demonstrating the potential of this class of drugs to act as anticancer agents [33]. Another recent study using different cell lines (malignant B, T, and myeloid cells) evaluated the effect of paroxetine and citalopram, two SSRIs, and found these drugs are able to decrease cancer cell proliferation and induce apoptosis, in a concentration-dependent manner, in a mechanism serotonergic-independent [34]. Nevertheless, the authors found these drugs were nonselective for cancer cells, warning of their use as standalone chemotherapeutic agents [34]; however, they also concluded these drugs could be used in tolerable doses, preferentially in combination with antineoplastic drugs, supporting their use as chemosensitizer agents [34]. Indeed, fluoxetine has been shown to inhibit the P-glycoprotein [35], a multidrug resistance extrusion pump involved in the development of cancer resistance, a feature that can be useful for adjuvant chemotherapy.

**Figure 3:**
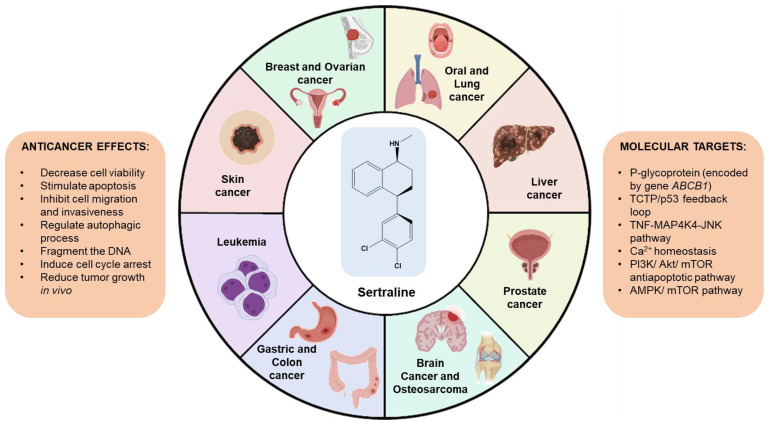
Overview of this review and main findings on the research of sertraline in cancer.

The discovery of the anticancer properties of sertraline by Adam Telerman and Robert Amson in 1993 was only the starting point of the many studies that urged, in the following years, the study of the anticancer effects of this repurposed drug [36]. Moreover, Telerman et al. were also responsible for the discovery of the involvement of the Translationally Controlled Tumor Protein (TCTP), a protein that is present in eukaryotic stem cells, in the process of tumor reversal [36]. This finding opened a new window of opportunities for the research of new drugs against this target for application in cancer therapy. As soon as the authors found this protein secreted histamine, they attempted to evaluate the use of antihistamines and other structurally similar molecules such as antipsychotics and antidepressants to inhibit TCTP. Among all drugs tested, sertraline was demonstrated to be the most promising inhibitor, causing an increase in the number of reversible clones in tumor lines by 30% [37]. Furthermore, in recent years, different case reports have suggested that the adjuvant use of sertraline is able to improve the clinical status of patients and even induce tumor remission [38,39]. After these findings, more and more studies on the anticancer effects of sertraline have been reported. The use of antidepressants as repurposable oncological drug candidates has already been reviewed recently [25]; nevertheless, no review was found on the exclusive anticancer effects of sertraline. Therefore, in this article, we focused on the review of the promising anticancer effects of sertraline against different human cancer cells. To perform this research, PubMed was used in September 2022 to investigate English papers using the terms “cancer AND sertraline”. The search was also restricted to the human species. In total, 129 records from PubMed were found. All papers from 1997 to date were scanned, and 26 were considered relevant for this review. The overview of this review is described in Figure 3.

## 2. Review on the Anticancer Effects of Sertraline against Different Human Cancer Cells

In the next sections, we will describe the main findings on the anticancer effect of sertraline against different types of cancer cells.

### 2.1. Lung Cancer

Lung cancer represents the leading cause of death by cancer worldwide, also being one of the most common types of cancer. Jiang and colleagues aimed to develop new therapies for lung cancer, especially in cases where patients develop resistance to tyrosine kinase inhibitors (TKIs) [40]. The authors used a medical genetics-based approach to investigate novel therapeutical uses for more than 1000 FDA-approved drugs and identified sertraline as a potential candidate with anticancer activity for the treatment of non-small cell lung cancer (NSCLC) [40]. They evaluated this antidepressant in vitro and found this drug is able to significantly decrease the viability of different TKI-resistant NSCLC cell lines (A549, H522, PC9/R, and H1975), with IC_50_ values 11.10, 10.50, 9.60, and 9.40 μM, respectively. Importantly, sertraline demonstrated synergic interactions with erlotinib, a cytostatic drug. Moreover, the authors found that the combined treatment of sertraline with erlotinib effectively promotes autophagy in these cells, as demonstrated by LC3-II accumulation and autolysosome formation [40]. Mechanistically, the combined treatment with sertraline and erlotinib affected the regulation of the AMP-activated protein kinase (AMPK)/the mechanistic target of rapamycin (mTOR) signaling pathway, as supported by the impairment in the anticancer effect of sertraline both alone and in combination with erlotinib after genetic knockdown of AMPK. Furthermore, the anticancer effect of this combination was found to decrease after pharmacological inhibition of autophagy or genetic knockdown of *ATG5* or *Beclin 1* [40]. Finally, the in vivo results using an orthotopic NSCLC mouse demonstrated that the combination of sertraline and erlotinib is able to successfully decrease tumor growth and increase mouse survival [40].

Recently, Zinnah et al. [41] evaluated if sertraline could sensitize A549, HCC-15, and Calu-3 human lung cancer cell lines to tumor necrosis factor-related apoptosis-inducing ligand (TRAIL). TRAIL has a significant role in cancer therapy due to its ability to eradicate tumoral cells without affecting non-tumoral cells; nevertheless, tumoral cells are commonly very resistant to TRAIL because they lack death receptor expression [41]. The authors have successfully demonstrated that sertraline inhibits autophagy and enhances the expression of death receptor 5 on TRAIL-resistant lung cancer cells. Specifically, the authors showed that sertraline induces TRAIL-mediated apoptosis by downregulating AMP-activated protein kinase phosphorylation, which results in the inhibition of autophagy and enhanced death receptor 5 expression, leading to activation of the apoptotic caspase cascade. Taken together, these results demonstrate that the repurposing of sertraline can be a promising strategy to increase the sensitization of lung tumoral cells to TRAIL [41].

### 2.2. Colorectal Cancer

Several studies have reported the importance of TCTP, a protein encoded by the gene *TCTP*, and its link with the sertraline anticancer effect in human cancer cells [42]. This protein is present in eukaryotic stem cells [43] and was first described in 1995, by Susan MacDonald [44]. It is also called the histamine-releasing factor due to its ability to release histamine [44]. Throughout the years, despite its histamine-release activity, it was also found this protein is involved in the maintenance of homeostasis, cell survival (through its anti-apoptotic action and involvement in cell stress pathways), cell cycle and development (it acts on microtubules and embryonic development). Furthermore, this protein regulates cell growth, protein synthesis, and degradation [45]. Moreover, TCTP also acts as a signaling molecule in immunological reactions [45].

A study compared the profiles of gene expression of revertant and parental tumor cells and found that TCTP has elevated expression in tumor cells, but barely undetected expression in revertant tumor cells [37]. Moreover, it was found that the silencing of TCTP in tumor cells could revert their malignant phenotype and even regulate apoptosis [37,42]. More recently, Amson et al. have described a negative feedback loop between TCTP and the tumor suppressor protein P53 [43]. They found that TCTP promotes the degradation of P53 via MDM2-mediated ubiquitination, inhibiting P53-mediated apoptosis and contributing to tumor growth [43]. On the other side, the authors also found that P53 can directly repress *TCTP* transcription. Therefore, they hypothesized that restoring P53 function in tumors by targeting the P53-MDM2 interaction could be a promising strategy for cancer combat. Hence, the authors aimed to find whether sertraline could interfere with the TCTP-dependent downregulation of P53. They found that sertraline increased the expression of P53 with a simultaneous reduction of TCTP expression, in HCT116 colon cancer cells [43]. In line with these results, the authors also found a dose-dependent decrease in the viability of HCT116 cells that were exposed to this drug. Furthermore, using *Trp53* knockdown HCT116 cells, it was found more resistance to sertraline in these cells than in wild-type HCT116 cells, demonstrating that these anticancer effects are dependent of P53. These results demonstrate that sertraline interferes with the TCTP-P53 feedback loop, by increasing the expression of P53 and decreasing the expression of TCTP, promoting P53-dependent apoptosis of cancer cells [43]. Moreover, it was found this drug also affects the self-renewal of cancer stem cells, as demonstrated by the reduction in the mammosphere-forming efficiency of primary mammary tumor cells from *ErbB2* transgenic mice [43]. Taken together, these results demonstrate that sertraline binds directly to TCTP, causing a reversion on the TCTP-induced ubiquitination of P53 and preventing the TCTP binding to MDM2, restoring the MDM2 auto-ubiquitination [43].

In 2008, Gil-Ad et al. [46] evaluated the cytotoxic effect of different antidepressants, including sertraline, on the viability and proliferation of two different human colorectal carcinoma cell lines: sensitive HT-29 and multi-drug resistant LS1034 cells. The authors demonstrated sertraline was able to significantly reduce the cell viability and proliferation in these cell lines in a dose-dependent manner, with IC_50_ values of 14.7 and 13.1 µM, respectively, for HT-29 and LS1034 cell lines. Furthermore, when comparing the anticancer effect of sertraline with common antineoplastic drugs like doxorubicin, vincristine, and 5-fluorouracil, it was found a similar anticancer effect in HT-29 cells and a superior cytotoxic effect in LS1034 cells [46]. Moreover, the authors also found sertraline treatment in HT-29 cells caused cell cycle arrest at G0/G1 and induced DNA fragmentation in a dose-dependent manner. Sertraline treatment also enhanced caspase-3 activation and increased c-Jun expression, causing a decrease in the expression of the anti-apoptotic protein Bcl-2, suggesting that sertraline may induce cell apoptosis through MAPK cascade activation and Bcl-2 inhibition. The in vivo studies using CD1 nude mice xenografted subcutaneously with HT-29 cells demonstrated that sertraline is able to significantly inhibit tumor growth after 5 weeks of treatment [46]. Taken together, the authors demonstrated that the antidepressant sertraline possesses anticancer activity against both sensitive and multi-drug resistant colon cancer cells and animal models.

**Figure 4:**
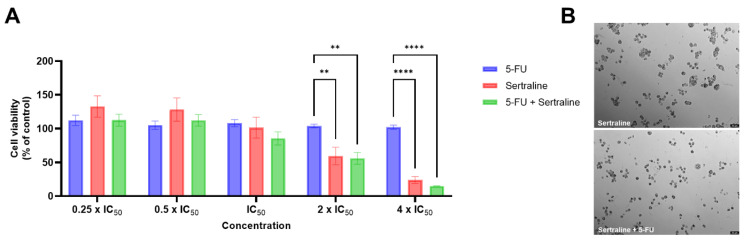
MTT results (**A**) and morphological analysis (**B**) of HT-29 cells treated for 48 h with a combination therapy of 5-FU and sertraline. Results are expressed in relation to control cells (treated with vehicle) and represent mean ± SEM. Each experiment was repeated three times (*n* = 3); ** statistically significant vs. control at *p* < 0.01. **** statistically significant vs. control at *p* < 0.0001. Scale bar: 50 µm. Reprinted with permission from Duarte et al. (2021). Copyright 2021 Copyright Duarte et al. [47].

Duarte et al. recently developed a new model of drug combination consisting of the antineoplastic drug 5-fluorouracil with different repurposed drugs, including sertraline, for colorectal cancer [47]. The authors treated HT-29 colon cancer cells with increasing concentrations (0.1–100 μM) of sertraline and found an IC_50_ of 2.45 μM, with a significant decrease in cell viability for all concentrations above 10 μM, resulting in more than 70% of cell death [47]. In the combination model, the experimental protocol for drug combinations was selected based on Chou-Talalay publications [48,49], where it is recommended to use constant-ratio drug combinations and to select several data points above IC_50_ and several below IC_50_, to make the determination of drug interactions more accurate. The combination of 2 and 4 times the IC_50_ value of 5-fluorouracil (6 and 12 µM, respectively) plus two and four times the IC_50_ value of sertraline (4.9 and 9.8 µM, respectively)_,_ resulted in enhanced significant toxicity compared to 5-fluorouracil alone (Figure 4) [47]. These concentrations are within the micromolar range and are considered acceptable for preclinical use. Moreover, this drug combination demonstrated strong synergism between the two drugs, which assures the potential of these drug combinations for dose optimization studies and further mechanistic studies [47].

Moreover, the authors also investigated the expression of some epithelial–mesenchymal transition biomarkers in HT-29 cells treated with 5-fluorouracil plus sertraline [50]. They found a more intense expression of E-cadherin in the combination treatments than compared to 5-FU and sertraline alone. It was also seen that this combination treatment leads to a smaller number of viable cells with less aggregation ability [50]. Regarding β-catenin, an intense expression of this protein in HT-29 cells treated with sertraline compared to control cells was found. Nevertheless, the authors found a decrease in the expression of this protein when cells were treated with 5-fluorouracil and sertraline combined. Vimentin expression also decreased upon treatment with sertraline alone and in combination. Taken together, these results demonstrate that sertraline, both alone and combined, could induce an epithelial–mesenchymal transition reversal [50].

Another study published by Duarte et al. [51] evaluated if honeybee venom could synergistically enhance the cytotoxic effect of several CNS agents, including sertraline, in HT-29 colorectal cancer cells [51]. Cells were treated with sertraline alone and in combination with increasing concentrations of bee venom for 48 h. The authors found that treatment with sertraline + honeybee venom caused a significant decrease in cell viability, both seen by morphological analysis and by MTT assay. Moreover, it was found that 6.25, 12.5 and 25 µg/mL of honeybee venom combined with sertraline produced better anti-cancer efficacy than each drug alone, supporting the use of sertraline in adjuvant strategies for colon cancer [51].

Although sertraline is reported to exert antineoplastic effects by inhibiting the serotonin reuptake transporters (SERT) activity, the anticancer mechanism and serotonin metabolism alterations in the absence of SERT have not been fully elucidated [52]. Recently, Ye et al. demonstrated that inhibiting SERT with sertraline promotes the uptake and catabolism of extracellular tryptophan in colon cancer, both in vitro and in vivo, in a mechanism that suppresses mTOR serotonylation, leading to mTOR inactivation and increased tryptophan uptake and catabolism, that increases serotonin biosynthesis and oncogenic metabolite kynurenine production [52]. Moreover, the authors also demonstrated that enhancing Trp metabolism promotes colon cancer growth. Therefore, they evaluated if the combination of SERT inhibition using sertraline with trametinib, a MEK inhibitor, could result in enhanced anticancer effects in colon cancer. The authors concluded that tryptophan uptake blockage can sensitize colon cancer cells to SSRIs such as sertraline [52].

### 2.3. Breast Cancer

Some reports demonstrated that some antidepressants can strongly inhibit CYP2D6 isoenzyme, affecting the concurrent use of other drugs, especially tamoxifen, a common antineoplastic agent used for breast cancer treatment [53]. Since sertraline has a less inhibitory potential of the CYP2D6 isoenzyme, it represents a preferential antidepressant agent for concomitant use with other drugs, especially in combination treatments of cancers in which tamoxifen is commonly used [53].

In 2010, Lin et al. [54] evaluated the anticancer effect of sertraline against MCF-7 breast cancer cells and found that this drug is able to significantly reduce cell viability, proliferation, and protein synthesis in a concentration-dependent manner. Moreover, the authors demonstrated that this drug is able to reduce polysome content and increase 80S ribosomes. Mechanistically, it was found that sertraline-induced inhibition in translation is related to a decrease in the eukaryotic initiation factor (eIF) 4F complex levels, altered localization of eIF4E, and increased eIF2α phosphorylation that leads to an increase in REDD1 expression, affecting the mTOR pathway [54]. This drug also targets the mTOR signaling pathway downstream of Rheb, independently. It was also found this drug is able to increase chemosensitivity to the chemotherapeutic agent doxorubicin in a murine lymphoma model [54].

Another study published by Bavadekar et al. (2014, [55]) evaluated seven antidepressants among the most prescribed in the US, including sertraline, against MCF-7 breast cancer cells and determined cell viability after a 24 h treatment using the XTT assay. The authors demonstrated that sertraline is cytotoxic against this cell line with an IC_50_ of 16 µM, ranking first in potency among the evaluated compounds [55]. They also demonstrated that sertraline induces apoptosis in these cells by analysis of morphological changes, phosphatidylserine externalization, and poly(ADP-ribose) polymerase (PARP) cleavage [55].

Gwynne et al. [56] recently found that different antagonists of serotonin biosynthesis and activity, including the antidepressant sertraline, target breast tumor-initiating cells in different breast cancer cell lines. Specifically, the authors demonstrated that sertraline affects breast tumor-initiating cells activity in a dose-dependent manner, by reducing the frequency of sphere-forming cells [56]. Furthermore, they also demonstrated that exposure of breast tumor cells ex vivo to sertraline induces a reduction in breast tumor-initiating cells frequency, decreases the growth rate of tumor allografts, and reduces their volume in a dose-dependent manner [56]. Moreover, the same research group also demonstrated that the combination of sertraline with docetaxel, a common antineoplastic used in the treatment of breast cancer, induces a reduction in tumor cell proliferation and causes cell death in mammary tumor allografts, in a synergistic way [57].

Geeraerts et al. [58] evaluated the use of two repurposed drugs, sertraline, and thimerosal, against serine/glycine synthesis-addicted breast cancer cancers using a yeast model system. The authors found that sertraline is able to inhibit the serine/glycine synthesis enzyme serine hydroxymethyltransferase. Furthermore, when combined with the mitochondrial inhibitor artemether, sertraline displayed an enhanced antiproliferative effect, caused by G1-S cell cycle arrest [58]. These findings were further supported by in vivo evidence, where it was found that this combination results in serine-selective antitumor activity in breast cancer mouse xenografts, which suggests that simultaneous inhibition of serine/glycine synthesis and mitochondrial metabolism can be used as a promising therapy for serine/glycine synthesis addicted cancers [58].

In another work, Duarte et al. investigated the anticancer effects of a new combination model consisting of chemotherapeutic agents commonly used in breast cancer therapy (paclitaxel and doxorubicin) and different repurposed drugs, including the antidepressant sertraline [47,50]. MCF-7 cells were treated with sertraline both alone and combined with paclitaxel and doxorubicin. At concentrations between 0.1 and 100 µM, sertraline alone demonstrated the ability to significantly reduce MCF-7 cell viability, at all concentrations above 10 µM, causing more than 70% of cell death, and with an IC_50_ of 2.22 µM, after a treatment of 48 h. When combined with paclitaxel, sertraline was not able to significantly increase the cytotoxic effect of the antineoplastic drug [47]. The combination of sertraline with doxorubicin also demonstrated that the use of sertraline as an adjuvant agent is not advantageous [50]. In the future, further studies are necessary to determine the mechanism of action of these combinations and the reasons behind the failure of these therapeutic regimens.

Recently, Duarte et al. evaluated if honeybee venom could synergistically be used in combined therapies, to enhance the cytotoxic activity of different CNS drugs, including sertraline, in the MCF-7 breast cancer cell line [51]. Cells were treated with sertraline, both alone and combined with increasing concentrations of bee venom, and it was found that sertraline significantly inhibits the growth of these cells, at concentrations above 10 µM. Moreover, the combination of sertraline and honeybee venom at the lowest concentration, demonstrated significant anticancer efficacy than both drugs alone, with more than 50% cell death, which supports the use of sertraline in adjuvant strategies for breast cancer [51].

### 2.4. Hepatocellular Cancer

A study published by Chen et al. (2014, [59]), investigated the anticancer effects of sertraline against the HepG2 human hepatocellular cancer cell line and found this drug is able to decrease cell viability and induce apoptosis in a time and dose-dependent manner. Mechanistically, they found this drug activates the intrinsic checkpoint protein caspase-9, inducing the release of cytochrome c from mitochondria to cytosol, in a process Bcl-2-dependent [59]. The sertraline-induced apoptosis was further investigated by pre-treating HepG2 cells with caspase-3, caspase-8, and caspase-9 inhibitors, which resulted in a significant decrease in lactate dehydrogenase release, indicating that sertraline induces apoptosis by intrinsic and extrinsic pathways [59]. The authors also found sertraline causes programmed cell death by increasing the expression of tumor necrosis factor (TNF) as well as the phosphorylation of JNK, ERK1/2, and p38, suggesting its anticancer effect may be related to the activation of the TNF-MAP4K4-JNK cascade signaling pathway [59].

Another study presented by Kuwahara et al. (2015, [60]) evaluated the cytotoxic effects of four different SSRIs, including sertraline, and two serotonin and norepinephrine reuptake inhibitors (SNRIs) against human hepatocellular carcinoma (HepG2) cells. They confirmed that sertraline is able to significantly decrease the viability of these tumor cells, with an IC_50_ of 1.24 µM [60]. Furthermore, the exposure to 2 µM sertraline caused a significant increase in the caspase-3/7 activity, demonstrating that HepG2 cells are sensitive to lower doses of sertraline and that the caspase signaling pathway may be involved in the mechanism of anticancer effects of sertraline [60].

### 2.5. Leukemia

Evidence on the proapoptotic and antiproliferative effects of SSRIs antidepressants on malignant lymphocytes has already been reported [61]. A study published by Amit et al. [61] evaluated the in vitro anticancer effects of different SSRIs in Jurkat T cell lymphoma leukemia cells, including sertraline. The authors found an IC_50_ value of 9.5 µM for sertraline in this cell line, with less than 40% of viable cells in concentrations above 15 µM. Using AlamarBlue™ viability assay, they also demonstrated that treatment of 48 h with this repurposed drug is able to significantly reduce cell viability in a higher degree than vincristine and cyclophosphamide, two commonly used antineoplastic drugs. Using the [^3^H] thymidine incorporation assay, they also evaluated the effect of sertraline in cell proliferation and found that this drug is able to decrease the number of proliferative cells [61]. In relation to apoptosis, an increase in the expression of caspase-3 and a decrease in the expression of Bcl-2 were found, with no alterations in the expression of key apoptotic proteins c-Jun and ERK. The authors also evaluated the effect of combining sertraline with vincristine and doxorubicin; they found an improvement in the anticancer effect of these chemotherapeutic drugs, suggesting that sertraline can act as a chemosensitizer in combination therapies [61].

In their work, Xia et al. [62] also demonstrated that sertraline has significant anticancer activity, not only in NB4, NB4-R1, and NB4-R2 acute myeloid leukemia (AML) cell lines, but also in the fresh leukemia cells isolated from AML patients. They also demonstrated that sertraline exerts cell death by two pathways: apoptosis and autophagy [62]. Furthermore, they demonstrated that autophagy inhibition leads to a reduction in sertraline-induced apoptosis and cell growth inhibition, indicating that the sertraline-induced autophagy process could be linked to AML cell apoptosis in some way. On the other hand, the inverse process of inhibiting apoptosis does not seem to impair sertraline-caused autophagy or cell growth inhibition [62].

### 2.6. Brain Cancer

Glioblastoma multiforme is a subtype of brain cancer that has a very aggressive phenotype and an extremely low life expectancy. Nordenberg et al. (2010, [63]) investigated the cytotoxic effects of novel drug combinations using conventional anticancer treatments (temozolomide or irradiation), imatinib (a targeted agent), and psychotropic drugs, including sertraline in human U87 glioblastoma cells. The authors found that sertraline alone is able to decrease cell content in a dose-dependent manner [63]. The combination of sertraline with temozolomide demonstrated an additive cytotoxic effect, while the combination of irradiation plus sertraline resulted in a less than additive effect. Interestingly, the combination of imatinib plus sertraline resulted in synergistic interactions [63]. Mechanistically, no changes were seen after single or combined treatments in the expression of phosphorylated MAPK. Nevertheless, a significant reduction in the expression of pAKT was detected after treatment of imatinib + sertraline, suggesting that the downregulation of pAKT may be involved in the synergist anticancer effect between imatinib and sertraline [63].

### 2.7. Melanoma

Melanoma is a type of cancer with a low response rate to chemotherapy and radiation. It is believed that the poor response to these therapeutic strategies may be related to the constitutive expression of Akt, which protects cancer cells from apoptosis. A study from 2008 published by Reddy et al. [64] aimed to evaluate the cytotoxic effects of sertraline against human melanoma cells and determine its mechanism of action in these cells. They demonstrated that sertraline can effectively reduce the cell viability of A375 human melanoma cells in a dose-dependent manner [64]. Moreover, by analyzing the expression of phosphorylated Akt, caspase 9, and phospho-p70 S6 kinase, the authors found that sertraline can effectively target Akt and inhibit its phosphorylation, inducing cell death through induction of the endoplasmic reticulum. Furthermore, in vivo studies in A375 xenografts demonstrated that sertraline is able to reduce tumor growth, proving that sertraline can act as an Akt inhibitor in melanoma [64].

Boia-Ferreira et al. (2017, [65]) also described the involvement of TCTP, an antiapoptotic protein that is overexpressed in several tumor types, including melanoma, in the cytotoxic activity of sertraline. Based on previous findings that demonstrated that sertraline interacts with TCTP and that inhibition of TCTP induces tumor reversion, in a reciprocal repression loop with P53, the authors studied the role of TCTP in melanoma using sertraline and siRNA. The authors concluded that treatment of MeWo, A2058, and B16-F10 melanoma cells with sertraline lead to the inhibition of TCTP causing a decrease in cell viability, reduction in migration properties, and diminished ability of cells to form colonies. Moreover, using a murine melanoma model, the authors also found that sertraline is able to significantly reduce tumor growth and at a higher degree than dacarbazine, an antineoplastic drug commonly used for the treatment of melanoma, demonstrating that this drug can effectively be repurposed for the treatment of skin cancer.

### 2.8. Oral Cancer

The regulation of cytosolic free Ca^2+^ levels is important for many pathophysiological responses in nearly all cell types, including tumor cells. There is evidence that, in some cases, an abnormal elevation in Ca^2+^ levels can induce apoptosis, protein dysfunction, proliferation, interference of ion flux, etc. Despite the existence of several studies that relate sertraline with the levels of Ca^2+^ in different types of cells, like smooth muscle cells and human platelets, its effect on cytosolic free Ca^2+^ levels in tumor cells, especially human oral cancer cells, is poorly understood. Based on this evidence, Chien et al. (2011, [66]) aimed to evaluate the effect of sertraline on the Ca^2+^ levels in human OC2 oral cancer cells. The authors used fura-2 as a Ca^2+^-sensitive fluorescent probe and found that, at a concentration range from 10 to 100 µM, sertraline caused an increase in cytosolic free Ca^2+^ levels, in a concentration-dependent manner [66]. Moreover, the signal decreased upon removal of extracellular Ca^2+^, suggesting that both Ca^2+^ entrance and release contribute to the rise in Ca^2+^ levels. Furthermore, the authors concluded that sertraline induced an increase in cytosolic free Ca^2+^ levels by causing phospholipase C-independent Ca^2+^ release from the endoplasmic reticulum and by Ca^2+^ influx via store-operated Ca^2+^ channels [66].

### 2.9. Ovarian Cancer

The development of drug resistance plays an important role in cancer therapy, being a major obstacle in the treatment of this disease. Specifically, P-glycoprotein and other multidrug-resistance-associated-proteins that belong to the ATP Binding Cassettes (ABC) transporter superfamily have been implicated in the development of drug resistance in cancer, since they are involved in the process of drug efflux from the cells [67]. Therefore, blocking these pump proteins seems to be a promising strategy for cancer therapy. Indeed, some reports have demonstrated that SSRIs can act as multiple drug resistance modulators and be effective chemosensitizers, both in vitro and in vivo. Therefore, Drinberg et al. [67] evaluated if sertraline could effectively be used as a multiple drug resistance modulator in vitro in the human ovarian adenocarcinoma cell lines OVCAR-8 and NCI/ADR-Res (NAR), a sub-line of OVCAR-8 expressing P-glycoprotein, and in vivo using human ovarian adenocarcinoma NCI-ADR/RES (NAR) xenografts. To do so, the authors combined sertraline with Doxil^®^ (also known as pegylated liposomal doxorubicin), to find if the combination would enhance the anticancer effect of Doxil^®^ while reducing side effects [67]. It was found that sertraline is able to modulate efflux pumps in cellular models of multidrug resistance. Moreover, the in vivo results demonstrated that the combination of sertraline with Doxil^®^ is efficient in reducing tumor growth and progression as well as extending the survival of tumor-bearing mice, proving the promising role of sertraline as a multidrug resistance modulator as well as a chemosensitizer in ovarian cancer [67].

### 2.10. Prostate Cancer

The effect of sertraline in the regulation of cytosolic free Ca^2+^ levels was further evaluated in prostate cancer cells by Huang et al. [68]. The authors aimed to evaluate if sertraline could alter the basal Ca^2+^ levels in PC-3 human prostate cancer cells. To do so, they used fura-2 as a Ca^2+^-sensitive fluorescent probe and found that, at a concentration range from 10 to 150 µM, sertraline induced an increase in cytosolic free Ca^2+^ levels, in a concentration-dependent manner [68]. They also found that the fluorescent signal decreased upon removal of extracellular Ca^2+^, suggesting that both Ca^2+^ entry and release contribute to the rise in Ca^2+^ levels. Moreover, the authors demonstrated that sertraline induced phospholipase C-dependent release of Ca^2+^ from the endoplasmic reticulum and from multiple Ca^2+^ influx pathways involving the store-operated Ca^2+^ channels. Finally, annexin V-FITC data showed that sertraline was also able to induce apoptosis in a concentration-dependent manner, independent of Ca^2+^ rise [68].

TCTP is an important survival factor of stem cells including cancer stem cells. Based on the findings that sertraline targets TCTP, Chinnapaka et al. aimed to investigate if sertraline could target prostate cancer stem cells [69]. The authors have demonstrated that sertraline efficiently inhibits tumorigenesis (assessed by colony growth assay), angiogenesis (evaluated by endothelial tube formation assay) and decreases the metastatic potential (tested using the wound healing and migration assays) of prostate cancer stem cells. Moreover, they also found that the cytotoxic effect of sertraline is dependent on oxidative stress, with sertraline inducing both apoptosis and autophagy, on the generation of free ROS, hydrogen peroxide formation, lipid peroxidation, and depletion of the levels of glutathione. Sertraline treatment also caused G0 arrest in prostate cancer stem cells [69]. Mechanistically, it was found that sertraline downregulates the expression levels of aldehyde dehydrogenase 1 and cluster of differentiation 44 (CD44) stem cell markers. Furthermore, sertraline also decreases the levels of TCTP, phospho TCTP, survivin, and cellular inhibitor of apoptosis protein 1; on the other side, this drug significantly increases the levels of cleaved caspase 3 and cleaved Poly [ADP-ribose] polymerase 1. It also affects the expression of stem cells, epithelial–mesenchymal transition, and autophagy markers, suggesting that sertraline can be successfully repurposed for the treatment of prostate cancer [69].

### 2.11. Gastric Cancer

Gastric cancer is a disease characterized by an elevated rate of morbidity. Although chemotherapy is still the main therapy for the treatment of the disease, the efficacy of the most common chemotherapeutic agents is still suboptimal, mainly due to the development of drug resistance [70]. Mu et al. investigated if the repurposed drug sertraline could act as a sensitizer of the drug-resistant gastric cancer cell line (SGC-7901/DDP). The authors found this drug successfully reduced the proliferation of this cell line, with an IC_50_ of 18.73 μM [70]. Moreover, they also synthesized 30 sertraline derivatives in an attempt to improve its cytotoxic activity and found an improved compound with an IC_50_ of 5.2 μM. Mechanistically, it was found that sertraline and its derivatives induced cell death by apoptosis and cell cycle arrest, and targeted the PI3K/Akt/mTOR signaling pathway [70].

### 2.12. Osteosarcoma

The effect of sertraline in the regulation of cytosolic free Ca^2+^ levels was also evaluated in MG63 human osteosarcoma cells by Lin et al. [71]. Here, the authors evaluated whether sertraline could change basal Ca^2+^ levels in a human osteosarcoma cell line. As previously described for similar studies, they used fura-2 as a Ca^2+^-sensitive fluorescent probe. The results are very similar to the ones described in Section 2.8 and Section 2.10. The authors found that, at concentrations from 50 to 200 µM, sertraline induced an increase in cytosolic free Ca^2+^ levels, in a concentration-dependent manner [71]. They also found that the Ca^2+^ signal diminished upon removal of extracellular Ca^2+^, indicating that both Ca^2+^ entry and release are involved in the increase of Ca^2+^ levels [71]. Moreover, the authors demonstrated that sertraline induced phospholipase C-dependent release of Ca^2+^ from the endoplasmic reticulum and Ca^2+^ entry by L-type Ca^2+^ channels and store-operated Ca^2+^ channels. Annexin V-FITC data showed that sertraline was also able to induce apoptosis in a concentration-dependent manner [71]. Treatment of this cell line with sertraline also leads to an increase in the levels of ROS, suggesting that cell death by apoptosis may involve mitochondrial pathways [71].

## 3. Discussion on the Repurposing of Sertraline for Cancer Therapy

The discovery of novel oncological therapies is an urgent research topic due to the low efficacy rates of the current cancer treatments. Drug repurposing offers the chance to discover novel therapeutical indications for drugs already existing in the market and used in clinical practice. This represents an advance over the development of new drugs as the repurposing process is much faster and requires less economical input, since repurposed drugs are already well-characterized, with all data regarding pharmacological and toxicological risks already available, increasing the chances of proceeding into clinical trials.

Sertraline is an antidepressant drug that belongs to the SSRIs and is mainly used for the management of major depressive disorder, obsessive–compulsive disorder, panic disorder, post-traumatic stress disorder, premenstrual dysphoric disorder, and social anxiety disorder [72]. Sertraline has primarily inhibitory effects on presynaptic serotonin reuptake. Serotonin plays an important role in regulating mood, personality, and wakefulness [72]. Therefore, blocking serotonin reuptake is beneficial in these types of disorders. Mechanistically, this drug blocks the pre-synaptic serotonin reuptake transporter SCL6A4, which inhibits the reuptake of serotonin, leading to its accumulation at the synaptic cleft [73,74]. The accumulation of serotonin at the synaptic cleft upregulates pre-synaptic 5-HT_1A_ auto-receptors, which results in a reduction of serotonin release by the pre-synaptic neuron [75,76]. Over time, prolonged exposure to elevated SSRIs concentrations leads to a desensitization of the pre-synaptic auto-receptors, which contributes to increasing the efficacy of treatment with this class of drugs [77,78]. Since patients with major depressive disorder have increased 5-HT_1A_ autoreceptor density [79,80], chronic treatment with SSRIs can contribute to desensitization of these receptors and therefore help in the management of these patients. Sertraline has also minimal effects on norepinephrine and dopamine uptake [81]. Despite its antidepressant effects, the discovery of sertraline as an inhibitor of TCTP and the involvement of this protein in tumor reversal has opened the path for novel studies on the anticancer activity of sertraline.

The aforementioned studies, summarized in Table 1, have investigated the antitumor effects of the antidepressant sertraline in different types of human cancer cells, and a vast majority have confirmed this drug has the potential to decrease the viability and proliferation of cancer cells, at concentrations of micromolar. These concentrations within the micromolar range are in line with most preclinical studies that evaluate drug cytotoxicity, and dose extrapolation from animals to humans has shown a therapeutic index of sertraline that could support future clinical trials. Moreover, these studies have demonstrated that sertraline induces apoptosis and regulates autophagy, and causes cell cycle arrest and DNA fragmentation. The regulation of autophagy is not completely understood and consensual, as some studies indicate sertraline induces autophagy, while others suggest that this drug inhibits the autophagic flux. It was also found that sertraline targets P-glycoprotein, SERT, and TCTP. TCTP, specifically, has an important role in the mechanism of action of sertraline in different types of cancer, such as colon, melanoma, and prostate, and is involved in the regulation of the expression of the tumor suppressor protein p53 in these types of cells. Moreover, it was found that sertraline activates different caspases, regulates ROS generation and Ca^2+^ levels, and decreases cell migration and invasion. In combination studies, it was found that sertraline can act as a chemosensitizer when combined with other agents such as tyrosine kinase inhibitors or antineoplastic drugs. This drug possesses anticancer activity both against sensitive and resistant cancer cell lines, being able to also inhibit the growth of cancer stem cells.

Mechanistically, it was found that sertraline can target several important signaling pathways, such as the TNF-MAP4K4-JNK, a pathway implicated in inflammatory and metabolic disorders and cancer progression, the PI3K/Akt/mTOR, involved in the regulation of cell growth, survival and metabolism, and the AMPK/mTOR, involved in protein synthesis and cell growth regulation. We hypothesize that sertraline may induce ROS production in cancer cells, which in turn alters the mitochondrial potential and induces DNA damage. This can lead to cell cycle arrest and fragmentation of damaged DNA, which causes cell death by apoptosis and an increase in Bax and caspases. Taken together, the previously mentioned studies support further in vivo studies and clinical trials.

Although the in vivo results described in this review suggest that sertraline decreases tumor growth and increases mouse survival, there is some controversy around the anticancer effects of sertraline, with some studies associating this drug with cancer-preventing effects, while others reported the opposite. Recently, Busby et al. [82] applied a novel combined connectivity mapping and pharmacoepidemiological strategy to identify drugs that affect breast cancer risk and found that sertraline was among cancer-preventing medications. In another population-based case–control study, Chan et al. [83] investigated the association between SSRIs use, including sertraline, and hepatocellular carcinoma risk. The authors found that sertraline use was associated with lower hepatocellular carcinoma risk [83]. On the other side, Christensen et al. [84] studied the effect of SSRIs, including sertraline, on survival and progression in ovarian cancer patients. They found that mice injected with sertraline have doubled mean tumor weight accompanied by an increase in the expression of the Ki67 marker, hypothesizing that sertraline treatment can alter serotonin levels in the tumor microenvironment, leading to the activation of proliferation pathways [84]. Nevertheless, in another study published by Morch et al. [85], in a nested case–control study of population-based registry data, the authors concluded that the use of SSRIs, including sertraline, was associated with a decreased risk of epithelial ovarian cancer, thereby suggesting a potential chemopreventive effect of this agent.

Nevertheless, and although most studies suggest the potential of sertraline for drug repurposing, the clinical use of sertraline in cancer therapy must take into account the side effects already described for its original indication. The most common side effects include nausea, diarrhea, constipation, vomiting, difficulty falling asleep or staying asleep, dry mouth, heartburn, loss of appetite, weight changes, dizziness, excessive tiredness, headache, nervousness, uncontrollable shaking of a part of the body, sexual problems and excessive sweating [86]. Therefore, the use of sertraline appears to cause side effects similar to other commonly used medications and seems to be compatible with clinical use. Nevertheless, taking sertraline long-term has been linked to an elevated risk of diabetes [87]. Care must also be taken regarding drug–drug interactions: sertraline interacts with monoamine oxidase inhibitors including isocarboxazid, linezolid, methylene blue, phenelzine, selegiline, tranylcypromine, and pimozide. It cannot be co-administered with disulfiram, and attention must be given to the administration of sertraline with other prescription and nonprescription medications, vitamins, nutritional supplements, and herbal products [88]. Therefore, clinicians should be cautious when prescribing this drug, with special attention to the development of side effects and drug–drug interactions that can occur upon treatment with sertraline. The risks and benefits of prescribing sertraline for cancer treatment should, therefore, be weighed against possible side effects guiding use. Furthermore, it is also important to develop novel strategies, such as nanoparticle formulations, that avoid the passage of sertraline over the blood–brain barrier while increasing its permeation into the tumors.

To date, two clinical trials concerning the repurposing of sertraline for cancer therapy are described in ClinicalTrials.gov: one proof-of-concept clinical trial assessing the safety of the coordinated undermining of survival paths by nine repurposed drugs, including sertraline, combined with metronomic temozolomide for recurrent glioblastoma, already completed (NCT02770378, [89]), and another aimed to determine the feasibility, safety, and toxicity of administering sertraline in combination with timed sequential cytosine arabinoside in adults with relapsed and refractory AML, without published results to date (NCT02891278, [90]).

## 4. Conclusions

In this review, we have provided solid evidence on the cytotoxic effect of sertraline against several types of cancer, supported by in vitro and in vivo results. Here, it was demonstrated that sertraline targets the P-glycoprotein, the TCTP protein as well as the TNF-MAP4K4-JNK, PI3K/Akt/mTOR, and AMPK/mTOR signaling pathways. This drug interferes with cell cycle regulation, affects DNA fragmentation, induces apoptosis, regulates autophagy, and causes a decrease in cancer cell viability, proliferation, migration, and invasion. Results in animal models suggest sertraline is a potent inhibitor of tumor growth, with the ability to decrease the metastasizing of cancer cells, demonstrating the anticancer potential of this antidepressant. Nevertheless, further research must be performed to develop novel formulations of sertraline to avoid the passage of this drug over the blood–brain barrier and concentrate the drug in tumor sites, as well as to decrease its side effects and drug–drug interactions. The potential of sertraline as an anticancer compound is further demonstrated by the ongoing clinical trials.

## Figures and Tables

**Table 1:** Summary of the main findings described in this review concerning the anticancer effects of sertraline. [+] and [-] indicate existence or absence of in vivo studies, respectively.

Condition	Cell Lines	In Vivo Results?	Main Findings	References
Lung cancer	A549, H522, PC9/R, and H1975	+	Sertraline is able to significantly decrease the viability of different TKI-resistant NSCLC cell lines. The combined treatment of sertraline with erlotinib effectively promotes autophagy. The combined treatment with sertraline and erlotinib affects the regulation of the AMPK/mTOR signaling pathway. The combination of sertraline and erlotinib is able to successfully decrease tumor growth and increase mouse survival.	[40]
	A549, HCC-15, and Calu-3	-	Sertraline induces TRAIL-mediated apoptosis by downregulation of AMP-activated protein kinase phosphorylation, which results in the inhibition of autophagic flux and upregulation of death receptor 5 expression, which leads to activation of the apoptotic caspase cascade.	[41]
Colorectal cancer	HCT116	+	Sertraline interferes with the TCTP-P53 feedback loop, by increasing the expression of P53 and decreasing the expression of TCTP, promoting P53-dependent apoptosis of cancer cells. This drug also affects the self-renewal of cancer stem cells, as demonstrated by the reduction in the mammosphere-forming efficiency of primary mammary tumor cells from *ErbB2* transgenic mice.	[43]
	HT-29 and LS1034	+	Sertraline was able to significantly reduce cell viability and proliferation in a dose-dependent manner. Sertraline treatment in HT-29 cells caused cell cycle arrest at G0/G1 and induced DNA fragmentation in a dose-dependent manner. Sertraline treatment enhanced caspase-3 activation and increased c-Jun expression, causing a decrease in the expression of the anti-apoptotic protein Bcl-2, suggesting that sertraline may induce cell apoptosis through MAPK cascade activation and Bcl-2 inhibition. Sertraline is able to significantly inhibit tumor growth after 5 weeks of treatment.	[46]
	HT-29	-	The combination of 5-fluorouracil with sertraline resulted in enhanced significant toxicity compared to 5-fluorouracil alone.	[47]
	HT-29	-	Sertraline, both alone and combined with 5-fluorouracil can induce an epithelial-mesenchymal transition reversal.	[50]
	HT-29	-	The combination of sertraline with honeybee venom produced better anti-cancer efficacy than each drug alone, supporting the use of sertraline in adjuvant strategies.	[51]
	SW480 and HCT116	+	Inhibiting SERT with sertraline promotes the uptake and catabolism of extracellular tryptophan in colon cancer, both in vitro and in vivo. Tryptophan uptake blockage can sensitize colon cancer cells to SSRIs such as sertraline.	[52]
Breast cancer	MCF-7	+	Sertraline significantly reduces cell viability, proliferation, and protein synthesis in a concentration-dependent manner. Sertraline reduces polysome content and increases 80S ribosomes. Sertraline-induced inhibition in translation is related to a decrease in the eukaryotic initiation factor (eIF) 4F complex levels, altered localization of eIF4E, and increased eIF2α phosphorylation that leads to an increase in REDD1 expression, affecting the mTOR pathway. Sertraline increases chemosensitivity to doxorubicin in a murine lymphoma model.	[54]
	MCF-7	-	Sertraline is cytotoxic against this cell line with an IC_50_ of 16 µM. Sertraline induced apoptosis in these cells, phosphatidylserine externalization, and PARP cleavage.	[55]
	BTIC	+	Sertraline affects breast tumor-initiating cells activity in a dose-dependent manner, by reducing the frequency of sphere-forming cells. Exposure of breast tumor cells ex vivo to sertraline induces a reduction in breast tumor-initiating cells frequency, decreases the growth rate of tumor allografts, and reduces their volume in a dose-dependent manner. The combination of sertraline with docetaxel induces a reduction in tumor cell proliferation and causes cell death in mammary tumor allografts, in a synergistic way.	[56,57]
	MDA-MB-231, MDA-MB-468, MCF-7 and HCC70	+	Sertraline inhibits the serine/glycine synthesis enzyme serine hydroxymethyltransferase. In combination with artemether, sertraline displayed an enhanced antiproliferative effect, caused by G1-S cell cycle arrest. This combination resulted in serine-selective antitumor activity in breast cancer mouse xenografts.	[58]
	MCF-7	-	Sertraline alone demonstrated the ability to significantly reduce cell viability, causing more than 70% of cell death, and with an IC_50_ of 2.22 µM. When combined with paclitaxel, sertraline was not able to significantly increase the cytotoxic effect of the antineoplastic drug. The combination of sertraline with doxorubicin demonstrated that the use of sertraline as an adjuvant agent is not advantageous.	[47,50]
	MCF-7	-	Sertraline significantly inhibits the growth of these cells, at concentrations above 10 µM. The combination of sertraline and honeybee venom demonstrated more significant anticancer efficacy than both drugs alone, with more than 50% cell death.	[51]
Hepatocellular cancer	HepG2	-	Sertraline is able to decrease cell viability and induce apoptosis in a time and dose-dependent manner. Sertraline activates the intrinsic checkpoint protein caspase-9, inducing the release of cytochrome c from mitochondria to cytosol, in a process Bcl-2-dependent. Sertraline induces apoptosis by intrinsic and extrinsic pathways. Sertraline causes programmed cell death by increasing the expression of tumor necrosis factor (TNF) as well as the phosphorylation of JNK, ERK1/2, and p38.	[59]
	HepG2	-	Sertraline was able to significantly decrease the viability of these tumor cells, with an IC_50_ of 1.24 µM. The exposure to 2 µM sertraline caused a significant increase in the caspase-3/7 activity.	[60]
Leukemia	Jurkat T cell	-	The IC_50_ value of 9.5 µM for sertraline, with less than 40% of viable cells in concentrations above 15 µM. Sertraline significantly reduces cell viability to a higher degree than vincristine and cyclophosphamide. Sertraline is able to decrease the number of proliferative cells. Sertraline increases the expression of caspase-3 and decreases the expression of Bcl-2. The combination of sertraline with vincristine and doxorubicin resulted in improvements in the anticancer effects.	[61]
	NB4, NB4-R1 and NB4-R2	+	Sertraline has significant anticancer activity. Sertraline exerts cell death by apoptosis and autophagy. Autophagy inhibition leads to a reduction in sertraline-induced apoptosis and cell growth inhibition.	[62]
Brain cancer	U87	-	Sertraline alone is able to decrease cell content in a dose-dependent manner. The combination of imatinib plus sertraline resulted in synergistic interactions. A significant reduction in the expression of pAKT was detected after treatment of imatinib + sertraline, suggesting that the downregulation of pAKT may be involved in the synergist anticancer effect between imatinib and sertraline.	[63]
Melanoma	A375	+	Sertraline can effectively reduce cell viability in a dose-dependent manner. Sertraline can effectively target Akt and inhibit its phosphorylation, inducing cell death through the induction of the endoplasmic reticulum. In vivo studies in A375 xenografts demonstrated that sertraline is able to reduce tumor growth.	[64]
	MeWo, A2058, and B16-F10	+	Treatment with sertraline leads to the inhibition of TCTP causing a decrease in cell viability, reduced migration properties, and a diminished ability of cells to form colonies. Sertraline was able to significantly reduce tumor growth to a higher degree than dacarbazine.	[65]
Oral cancer	OC2	-	Sertraline causes an increase in cytosolic free Ca^2+^ levels, in a concentration-dependent manner. Sertraline induces an increase in cytosolic free Ca^2+^ levels by causing phospholipase C-independent Ca^2+^ release from the endoplasmic reticulum and by Ca^2+^ influx via store-operated Ca^2+^ channels.	[66]
Ovarian cancer	OVCAR-8 and NCI/ADR-Res (NAR)	+	Sertraline is able to modulate efflux pumps in cellular models of multidrug resistance. In vivo results demonstrated that the combination of sertraline with Doxil^®^ is efficient in reducing tumor growth and progression as well as extending the survival of tumor-bearing mice.	[67]
Prostate cancer	PC-3	-	Sertraline induces an increase in cytosolic free Ca^2+^ levels, in a concentration-dependent manner. Sertraline induces phospholipase C-dependent release of Ca^2+^ from the endoplasmic reticulum and from multiple Ca^2+^ influx pathways involving the store-operated Ca^2+^ channels. Sertraline was also able to induce apoptosis in a concentration-dependent manner, independent of Ca^2+^ rise.	[68]
	PCSC	-	Sertraline efficiently inhibits tumorigenesis, angiogenesis and decreases the metastatic potential of prostate cancer stem cells. Sertraline induces both apoptosis and autophagy by the generation of free ROS, hydrogen peroxide formation, lipid peroxidation, and depletion of the levels of glutathione. Sertraline causes G0 arrest in prostate cancer stem cells. Sertraline downregulates the expression levels of aldehyde dehydrogenase 1 and cluster of differentiation 44 (CD44) stem cell markers. Sertraline decreases the levels of TCTP, phospho TCTP, survivin, and cellular inhibitor of apoptosis protein 1; on the other side, this drug significantly increases the levels of cleaved caspase 3 and cleaved Poly [ADP-ribose] polymerase 1. Sertraline affects the expression of stem cells, epithelial–mesenchymal transition, and autophagy markers.	[69]
Gastric cancer	SGC-7901/DDP	-	Sertraline reduces the proliferation of this cell line. Sertraline and its derivatives induced cell death by apoptosis and cell cycle arrest and targeted the PI3K/Akt/mTOR signaling pathway.	[70]
Osteosarcoma	MG63	-	Sertraline increases cytosolic free Ca^2+^ levels, in a concentration-dependent manner. Sertraline induces phospholipase C-dependent release of Ca^2+^ from the endoplasmic reticulum and Ca^2+^ entry by L-type Ca^2+^ channels and store-operated Ca^2+^ channels. Sertraline induces apoptosis in a concentration-dependent manner. Treatment with sertraline also leads to an increase in the levels of ROS, suggesting that cell death by apoptosis may involve the mitochondrial pathway.	[71]

## Data Availability

Not applicable.
